# Formulation and Optimization of Sustained Release Stavudine Microspheres Using Response Surface Methodology

**DOI:** 10.5402/2011/627623

**Published:** 2011-06-28

**Authors:** Sanjay Dey, Soumen Pramanik, Ananya Malgope

**Affiliations:** ^1^Department of Pharmaceutical Sciences, Dibrugarh University, Dibrugarh, Assam 786004, India; ^2^Department of Pharmaceutics, Royal College of Pharmacy & Health Sciences, Andhapasara Road, Berhampur, Orissa 760002, India

## Abstract

The aim of the current study was to formulate and optimize the formulation on the basis of *in vitro* performance of microsphere. A 3^2^ full factorial design was employed to study the effect of independent variables, polymer-to-drug ratio (*X*_1_) and stirring speed (*X*_2_), on dependent variables, encapsulation efficiency, particle size, and time to 80% drug release. The best batch exhibited a high entrapment efficiency of 70% and mean particle size 290 *μ*m. The drug release was also sustained for more than 12 hours. The study helped in finding the optimum formulation with excellent sustained drug release.

## 1. Introduction

Microencapsulation is a useful method for prolonging drug release from dosage form and reducing adverse effect [1]. Recently, dosage forms that can precisely control the release rates and target drugs specific body site have made an enormous impact in the formulation and development of novel drug delivery system. Microspheres form an important part of such novel drug delivery system [2–4]. Microspheres are one of the multiparticulate delivery systems and are prepared to obtain prolonged or controlled drug delivery, to improve bioavailability or stability and to target drug to specific sites. Microspheres can also offer advantages like limiting fluctuation within therapeutic range, reducing side effects, decreasing dosing frequency, and improving patient compliance [5, 6].

Ethylcellulose (EC) that is a hydrophobic and pH-independent polymer has been widely used in the prepared sustained release dosage forms of a water-soluble material [7–10]. The substance encapsulated in the microsphere is released under the influence of a specific stimulus at a specified stage [11]. Whereas interaction between dissolution media, polymer, and drug is the primary factors in release control [12, 13], various formulation variables influence drug release rate to greater or lesser extent. Thus, drug loading [14, 15], drug/polymer ratio [16–18], and drug particle size [19] have been shown to affect drug release from EC matrices. 

Stavudine is a nucleotide reverse transcriptase inhibitors and primarily used in the treatment of one of the most common chronic disease of the planet, AIDS. It has short biological half-life 0.8–1.5 h, and low daily dose of 30 mg is required [20, 21].The frequency of dosing is more. To overcome this problem it is necessary to desire sustained release dosage forms to improve patient compliance. 

Response surface methodology (RSM) is widely practiced approach in the development and optimization of drug delivery devices. Based on the principle of design of experiments (DoEs), the methodology encompasses the use of various types of experimental designs, generation of polynomial equations, and mapping of the response over the experimental domain to determine the optimum formulation(s) [22–27]. The technique requires minimum experimentation and time, thus proving to be far more effective and cost-effective than the conventional methods of formulating dosage forms.

The current study aims at developing and optimizing microspheres of stavudine using RSM, as it may prove to be more productive than the conventional systems by virtue of prolongation of drug residence time in gastrointestinal tract. Further, microsphere of the drug would involve relatively more economical and less complicated technology vis-à-vis many other drug delivery devices. Computer-aided optimization technique, using a full factorial design, was employed to investigate the effect of 2 independent variables (factors) (i.e., the drug-to-polymer ratio and stirring speed) on particle size, encapsulation efficiency, and drug release.

## 2. Materials and Methods

### 2.1. Materials

Stavudine was obtained from Cipla Ltd, Mumbai, India as gift sample. EC was procured from CDH (P) Ltd, New Delhi, India. Acetone and light liquid paraffin were obtained from Ranbaxy Fine Chemical Ltd., New Delhi, India used as dispersion media. n-Hexane (Ranbaxy Fine Chemical Ltd., New Delhi, India) was a washing agent. All chemicals received were of analytical grade and were used as such.

### 2.2. Methods

Microspheres of ethylcellulose were prepared by emulsion solvent diffusion technique, using EC as a polymeric retardant material. Polymer was dissolved in 5 mL organic solvent consisting of acetonitrile and dichloromethane (1 : 1 ratio). The resultant solution was extruded through a syringe (G 20) into the solution of drug in aqueous medium (2 mL) under stirring at 500 rpm using mechanical stirrer (Remi Motors, India) for 5 minutes, to form primary emulsion (w/o). The w/o primary emulsion was slowly added to 50 mL of light liquid paraffin containing 0.5% span-80, 1% ethylcellulose (50 mg), 1% w/v magnesium stearate (50 mg) as a tensioactive agent, saturation, and droplet stabilizer in the processing medium, respectively, under stirring in different rpm to form w/o/o multiple emulsion.The whole system was stirred for about 3 h. After stirring process was over, the light liquid paraffin was decanted off and microspheres formed were collected by filtration using ordinary filter paper (pore size 25 *μ*m) and treated with petroleum ether (40–60°C) for several times to completely remove the oil. Microspheres then air dried at room temperature for 12 h and collected for further studies. The drug-to-polymer ratio and stirring speed were varied in batches F1 to F9.

### 2.3. Assay of Stavudine

Stavudine was estimated by ultraviolet visible (UV/Vis) spectrophotometric method (Hitachi, U-1700, Japan). Aqueous solution of stavudine was prepared in phosphate buffer (pH 6.8), and absorbance was measured on UV/Vis spectrophotometer at 266 nm. The method was validated for linearity, accuracy, and precision. The method obeys Beer's Law in the concentration range of 5 to 50 *μ*g/mL. When a standard drug solution was analyzed repeatedly (*n* = 6), the mean error (accuracy) and relative standard deviation (precision) were found to be 0.7% and 1.2%, respectively.

### 2.4. Drug Entrapment Efficiency

Microspheres (50 mg) were crushed in a glass mortar and pastle, and the powdered microsphere were suspended in 50 mL phosphate buffer (pH 6.8). The resulting mixture was shaken by the magnetic stirrer for 24 h. The solution was filtered, and the filtrate was analyzed for the drug content. The drug entrapment efficiency was calculated using the following formula:

(1) $$\begin{array}{cc} & \text{Drug}\;\text{Entrapment}\;\text{Efficiency}\\ & \;=\frac{\text{Practical}\;\text{drug}\;\text{content}}{\text{Theoritical}\;\text{drug}\;\text{content}}\times 100. \end{array}$$

### 2.5. Particle Size Analysis

Particle size of the microspheres was measured by laser light scattering technique (Mastersizer 2000, Malvern, UK). The sizes of the completely dried microspheres of different formulations were measured by dry sample technique using dry sample adapter. The completely dried particles were placed on the sample tray with an in-built vacuum, and compressed air system was used to suspend the particles. The laser obscuration range was maintained between 1% and 2%. The volume mean diameter (*V*_*d*_) was recorded. After measurement of particle size of each sample, the dry sample adopter was cleaned thoroughly to avoid cross contamination. Each batch was analyzed in triplicate, but the average values were considered in data analysis.

### 2.6. *In Vitro* Drug Release Study

*In vitro* drug release study of microspheres were carried out using USP XXIV paddle type apparatus (Campbell Electronic, Mumbai, India) at 37 ± 1°C and at 100 rpm using 900 mL phosphate buffer (pH 6.8). Microsphere equivalent to 50 mg of stavudine were used for the test. At predetermined intervals, 5 mL of aliquots were withdrawn and replaced by the same volume of fresh media. Aliquots were filtered through a 0.45 *μ*m membrane filter, diluted suitably, and analysed spectrophotometrically. Percent of drug dissolve at different time intervals was calculated using Lambert-Beer's equation (*Y* = 0.0463*X* − 0.006) describe above. The *t*_80%_ was calculated using the Weibull equation [24]. The samples were then filtered through membrane filter (0.45 *μ*m) and diluted suitably. The amount of drug present in the solution was then analyzed spectrophotometrically at 225 nm using UV-Visible spectrophotometer (Shimadzu, UV-1700, Japan).

### 2.7. Scanning Electron Microscopy (SEM) Study

The surface topography of the prepared microspheres was examined by scanning electron microscope (Hitachi, S-3600N, Japan). The samples were fixed on brass stub using double-sided tape and then gold-coated in vacuum by a sputter coater. The SEM pictures were then taken at an excitation voltage of 20 kV.

### 2.8. Factorial Design

A statistical model incorporating interactive and polynomial terms was used to evaluate the responses:

(2) $$Y=b_{0}+b_{1}X_{1}+b_{2}X_{2}+b_{12}X_{1\;}X_{2}+b_{11}X_{1}^{2}+b_{22}X_{2}^{2},$$

where *Y* is the dependent variable, *b*_0_ is the arithmetic mean response of the 9 runs. The main effects (*X*_1_ and *X*_2_) represent the average result of changing one factor at a time from its low to high value. The interaction terms (*X*_1_ *X*_2_) show how the response changes when 2 factors are simultaneously changed. The polynomial terms (*X*_1_^2^  and  *X*_2_^2^) are included to investigate nonlinearity. 

## 3. Results and Discussion

Microspheres of stavudine were prepared by emulsion solvent diffusion technique using EC as a polymer due to its hydrophobicity and release-controlling properties. At first in trial batch, viscosity of the polymer solution is optimized since it is an important factor related to microspheres as reported by Lee et al. [28]. Polymer concentration of 0.5%, 1%, and 2% w/v were selected for preliminary trials. Flake formation was observed when ethylcellulose concentration was used at a level of 0.5%, whereas maximum sphericity was observed at the 1% level. The ethylcellulose solution was found to be too viscous to pass through the syringe when used at the 2% level. Therefore, 1% was found to be the optimum concentration for the entire factorial batch. 

The volume of secondary oil phase is an important factor as related to the formulation of microspheres. The volume of light liquid paraffin to be considered for the factorial batch was selected on the basis of *in vitro* release study. Different volume of light liquid paraffin (from 20 to 60 mL) was used in trial batch. As the volume of light liquid paraffin is increased from 20 mL to 50 mL, the *in vitro* release of stavudine is significantly (*P* < .05) decreased. On the other hand, insignificant (*P* > .05) decrease of the* in vitro *drug release was observed when the light liquid paraffin volume was increased from 50 mL to 60 mL. Concentration of span-80 plays an important role in the formulation of microspheres prepared by emulsion solvent diffusion technique. Therefore, suitable concentration of span-80 for factorial batch was selected by taking into account their aggregation phenomenon. Span-80 in the concentration of 0.5% was found suitable to prevent aggregation of the microspheres. 

**Figure 1:**
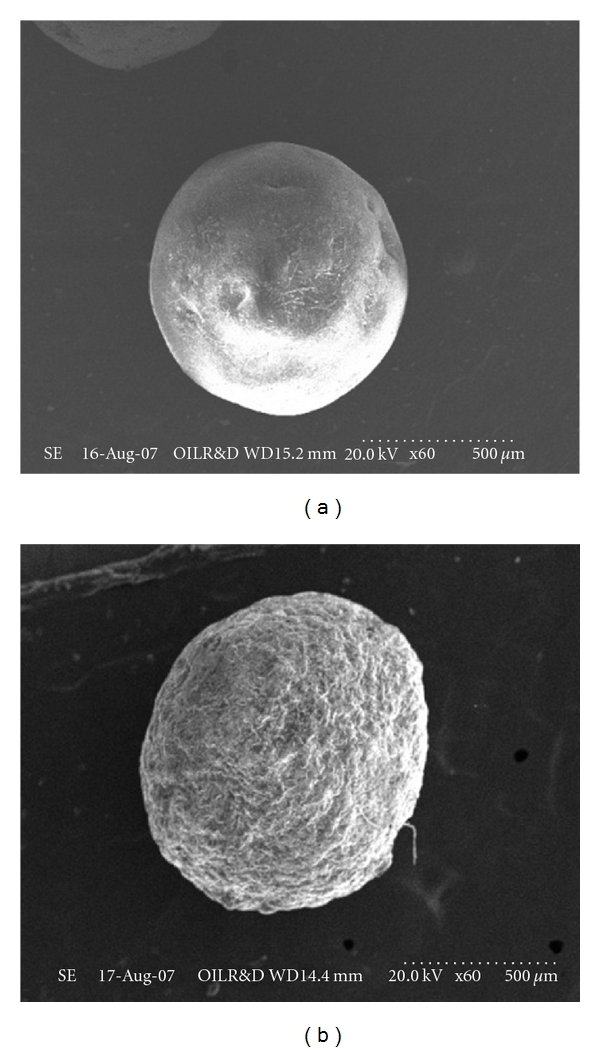
SEM drug-loaded ethylcellulose microsphere (a) before dissolution and (b) after dissolution.

The SEM photograph (Figure 1) revealed that the drug-loaded microspheres are spherical. Microspheres prepared containing higher amount of the polymer (1 : 3 drug : polymer ratio) exhibited smoother surfaces than those prepared taking a lower amount of the polymer (1 : 1 and 1 : 2). Irregular surfaces and larger sizes of the microspheres were observed for those prepared with a lower amount of the polymer. This has greatly affected the morphological characteristics of the microspheres. As the drug-to-polymer ratio was increased, more spherical microspheres with smooth surfaces were obtained as suggested earlier [29]. 

On the basis of the preliminary trials a 3^2^ full factorial design was employed to study the effect of independent variables (i.e., drug-to-polymer ratio [*X*_1_] and the stirring speed [*X*_2_]) on dependent variables (mean particle size, drug entrapment efficiency, and *t*_80%_). The results depicted in Table 1 clearly indicate that all the dependent variables are strongly dependent on the selected independent variables as they show a wide variation among the 9 batches (B1 to B9). The fitted equations (full models) relating the response (i.e., mean particle size, drug entrapment efficiency, and *t*_80%_) to the transformed factor are shown in Table 2. The polynomial equations can be used to draw conclusions after considering the magnitude of coefficient and the mathematical sign it carries (i.e., positive or negative). The high values or correlation coefficient (Table 2) for the dependent variables indicate a good fit.

Mathematical relationship generated using multiple linear regression analysis for the studied variables are expressed as follows:

(3) $$\begin{array}{cc} & \text{Mean}\;\text{particle}\;\text{size}\\ & \;=244.38+32.83X_{1}-8.83X_{2}-1.50X_{1}X_{2}\\ & \;\;+1.67X_{1}^{2}-0.33X_{2}^{2}, \end{array}$$

(4) $$\begin{array}{cc} & \text{Entrapment}\;\text{efficiency}\\ & \;=59.07+8.33X_{1}-3.50X_{2}+0.50X_{1}X_{2}\\ & \;\;-1.24X_{1}^{2}+1.26X_{2}^{2}, \end{array}$$

(5) $$\begin{array}{cc} t_{80\%} & =348.79+124.33X_{1}-19.67X_{2}-24.00X_{1}X_{2}\\ & \;+79.72X_{1}^{2}-42.28X_{2}^{2}. \end{array}$$

All the polynomial equations were found to be statistically significant (*P* < 0.01), as determined using ANOVA (Table 3), as per the provision of Design Expert software.

**Figure 2:**
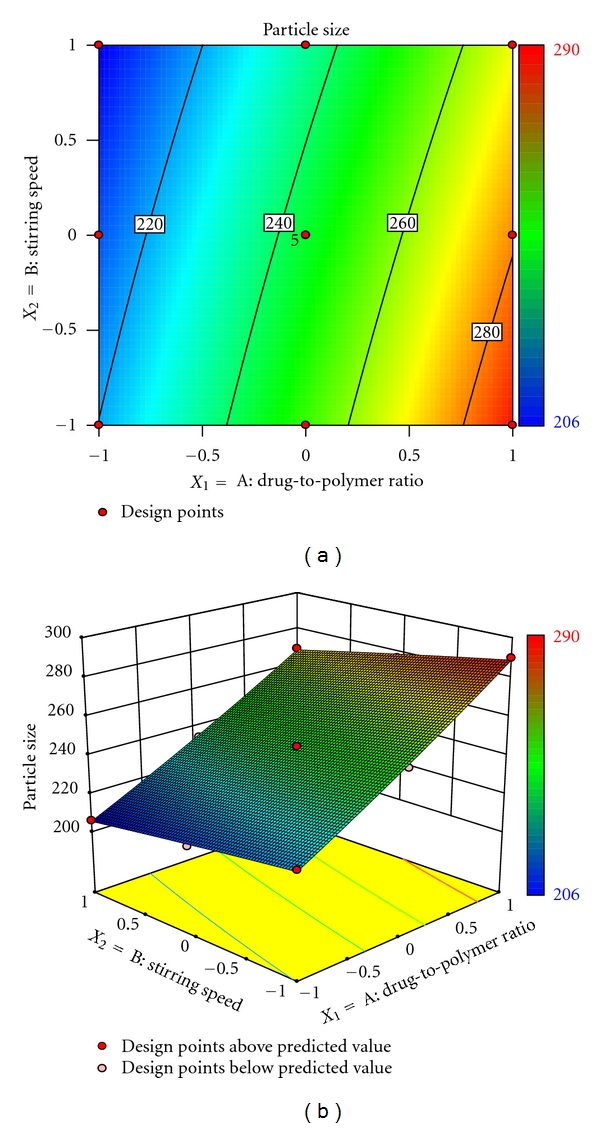
(a) Response surface plot showing the influence of drug-to-polymer ratio and stirring speed on mean particle size (*μ*m) and (b) corresponding contour plot showing the relationship between various levels of 2 independent variables.

Particle size distribution was found to be unimodal. Particle size analysis of microspheres was found to be in the range of 206–290 *μ*m (Table 1). The factorial equation for particle size showed good correlation coefficient (0.9979). Results of the equation indicate that the effect of *X*_1_ (drug-to-polymer ratio) is more significant than *X*_2_ (stirring speed). Moreover, stirring speed had a negative effect on the particle size (i.e., as the stirring speed increased, the particle size decreased). As the stirring speed was increased, the size of microdroplet of the emulsion was decreased resulting in the formation of smaller size microparticles. These findings are similar to those reported previously (27,28). Figures 2(a) and 2(b) depict a linear trend of mean particle size in an ascending order with an increase in each variable. It also shows that drug-to-polymer ratio has a comparatively greater influence on the response variables than stirring speed.

**Figure 3:**
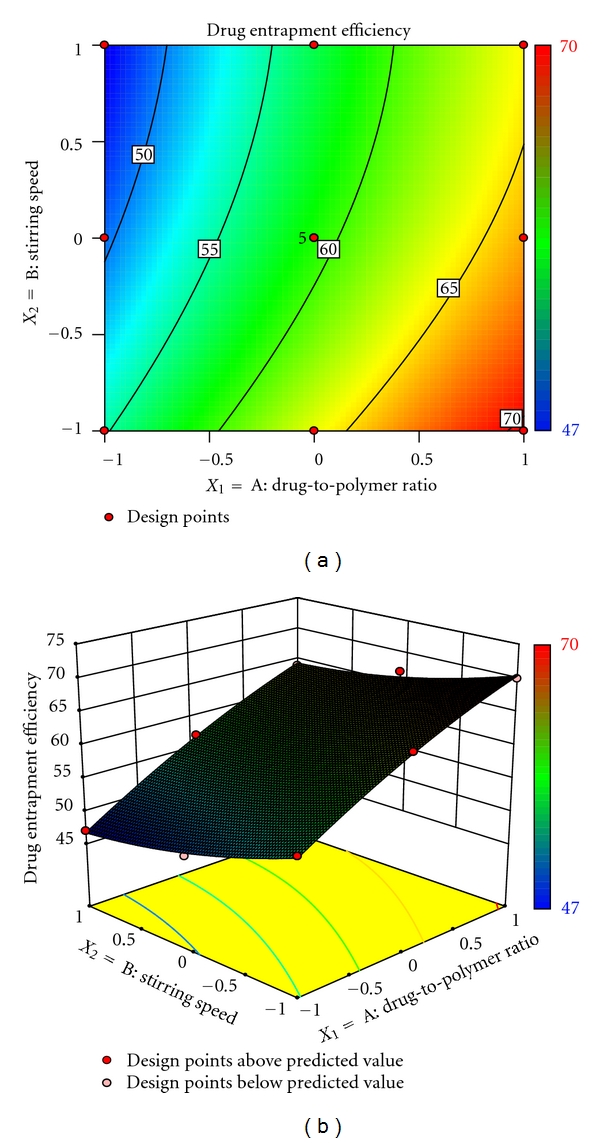
(a) Response surface plot showing the influence of drug-to-polymer ratio and stirring speed on entrapment efficiency (%) and (b) corresponding contour plot showing the relationship between various levels of 2 independent variables.

The drug entrapment efficiency is important variable for assessing the drug loading capacity of microspheres. This parameter is dependent on the process of preparation, physicochemical properties of drug, and formulation variables. The drug entrapment efficiency varied from 47% to 70% and showed good correlation coefficient (0.9970). Result of equation indicates the effect of *X*_1_ (drug-to-polymer ratio) is more significant than *X*_2_ (stirring speed). Moreover, stirring speed had a negative effect on drug entrapment efficiency (i.e., the stirring speed increased, the particle size decreased, and thus drug entrapment efficiency decreased). As the ratio of drug-to-polymer increased, encapsulation efficiency increased; this is due to the fact that higher ratio of drug-to-polymer would produce large size droplets with decreased surface area, such that diffusion of drug from such microsphere will be slow, resulting in higher encapsulation efficiency. Figures 3(a) and 3(b) also exhibit that entrapment efficiency vary in a nonlinear manner, but in ascending pattern with an increase in each variable. But at higher level of stirring speed the contour lines tend to be linear. However, the effect of drug-to-polymer seems to be more pronounced as compared to stirring speed.

**Figure 4:**
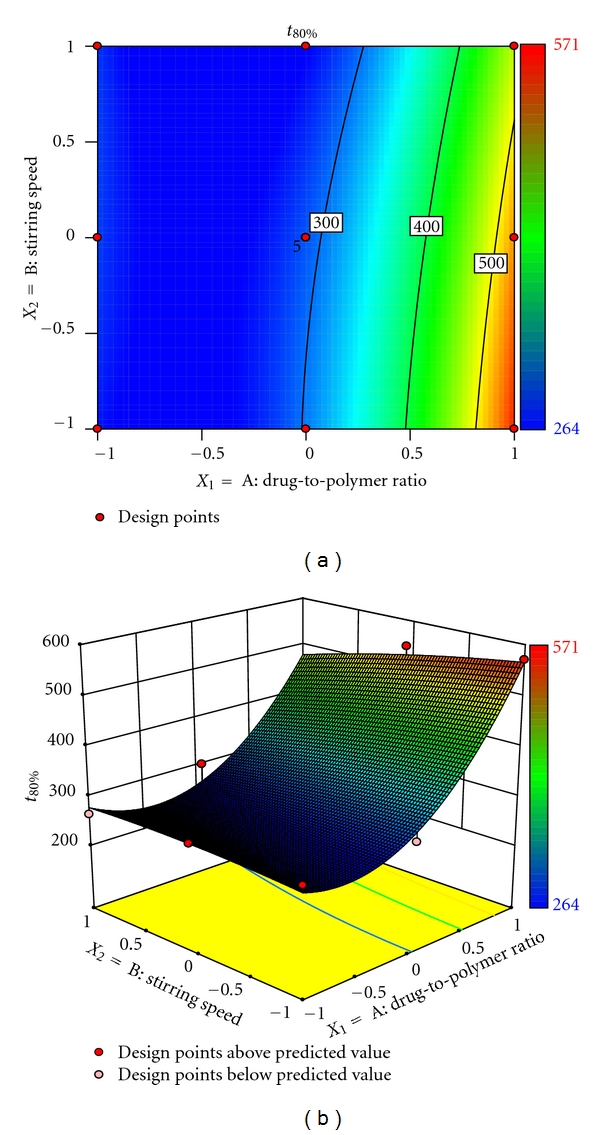
(a) Response surface plot showing the influence of drug-to-polymer ratio and stirring speed on *t*_80%_ (minutes) and (b) corresponding contour plot showing the relationship between various levels of 2 independent variables.

**Figure 5:**
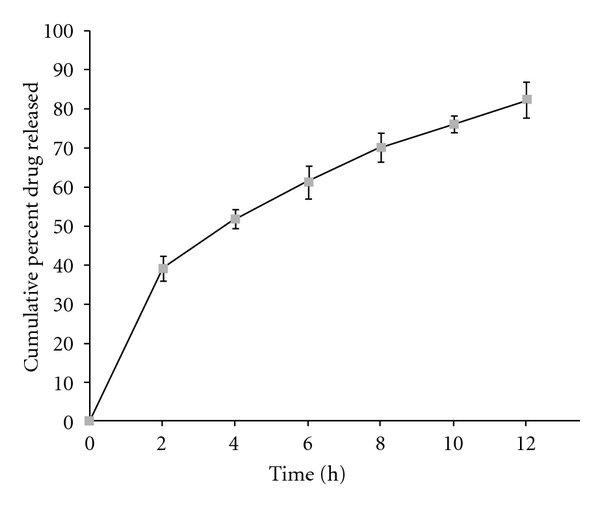
*In vitro* release profile of stavudine from ethylcellulose microspheres of batch F7.

The release profiles of formulations appear to be slow release with negligible burst effect. The formulations with lower levels of drug-to-polymer ratio exhibited higher initial burst in drug release. This could be attributed to the dissolution of drug present initially at the surface of the microspheres. However, the formulations showed little burst effect at higher drug-to-polymer ratio, ratifying better sustenance of drug released. The values of *t*_80%_ enhanced markedly from 264 minutes, observed a low levels of drug-to-polymer ratio and stirring speed, to as high as 571 minutes, observed at high level of drug-to-polymer ratio and stirring speed. This finding indicated considerable release-retarding potential of the polymers for stavudine. Batch F7 exhibited a high *t*_80%_ of 517 minutes and since it is a promising candidate for achieving drug release up to 12 hrs. The drug release profile of batch F7 is shown in Figure 5. The percentage *in vitro* drug release is highly dependent on drug-to-polymer ratio and stirring speed. Results depicted in Table 2 indicate that the effect of drug-to-polymer ratio (*X*_1_) is more significant than stirring speed (*X*_2_). The stirring speed had a negative effect on *t*_80%_ because as the stirring speed increased, the particle size of microspheres is increased, resulting in decrease of drug release. On the other hand, as the drug-to-polymer ratio is increased, the drug loading was decreased, resulting in decrease of drug release from microspheres. On the other way, increase in polymer matrix into the microspheres leads to an increased diffusional path length and thereby decreased the overall drug release from microspheres. Furthermore, smaller microspheres are formed at lower polymer concentration and have larger surface area exposed to dissolution medium. Figures 4(a) and 4(b) show that *t*_80%_ vary in nonlinear fashion, but in ascending pattern with an increase of each variable. The contour plot (Figure 5(b)) shows that drug-to-polymer ratio has a comparatively greater influence on the response variable than stirring speed.

The optimum formulation was selected based on the criteria of attaining complete and controlled release with highest possible entrapment efficiency. Upon “trading off” various response variables, the following maximizing criteria were adopted: mean particle size <300 *μ*m, entrapment efficiency >60%, and *t*_80%_ > 540 minutes. Accordingly, formulation F7 was ranked as best batch. In order to determine the mechanism of drug release from the formulation F7, the data obtained *in vitro* release study were fitted to the Korsemeyer-Peppas model in order to determine the “*n*” value, which describes the drug release mechanism. The “*n*” value of all the formulations was between 0.5 and 1 indicating that the mechanism of drug release was non-Fickian type diffusion.

## 4. Conclusion

Sustained drug release in the current study indicates that the hydrophobic matrix microspheres of stavudine, prepared using EC, can successfully be prepared by emulsion solvent diffusion technique. The results of a 3^2^ factorial design revealed that the drug-to-polymer ratio and stirring speed are imperative to acquire sustained release and entrapment efficiency. The microspheres of best batch exhibited mean particle size of 290 *μ*m and entrapment efficiency of 70%. The *t*_80%_ of 571 minutes indicates that the microspheres of stavudine could sustain the release of the drug for more than 12 h.

## Figures and Tables

**Table 1:** 3^2^ full factorial design layout.

Batch code	Variable levels in coded Form		Particle size (*μ*m)*	Drug entrapment efficiency (%)*	*t*_80%_ (minutes)*
	*X*_1_	*X*_2_			
F1	−1	−1	221	55	283
F2	−1	0	212	49	281
F3	−1	1	206	47	264
F4	0	−1	251	64	284
F5	0	0	245	59	287
F6	0	1	234	57	300
F7	1	−1	290	70	571
F8	1	0	277	67	547
F9	1	1	269	64	456

Translation of coded levels in actual units					
Variables level		Low (−1)		Medium (0)	High (+1)

Drug-to-polymer ratio (*X*_1_)		1 : 1		1 : 2	1 : 3
Stirring speed (*X*_2_) rpm		800		1000	1200

*Average of three determination.

**Table 2:** Summary of results of regression analysis.

Coefficient	*b*_0_	*b*_1_	*b*_2_	*b*_11_	*b*_22_	*b*_12_	*R^2^*
Entrapment efficiency (%)	59.07	8.33	−3.50	0.50	−1.24	1.26	0.9970
Mean particle size (*μ*m)	244.38	32.83	−8.83	−1.50	1.67	−0.33	0.9979
*t*_80%_ release (min)	348.79	124.33	−19.67	−24.00	79.72	−42.28	0.9694

**Table 3:** Analysis of variance (ANOVA) for all three responses^(a)^.

	Entrapment efficiency (%) (*Y*_1_)		Particle size (*μ*m) (*Y*_2_)		*t*_80%_ release (min) (*Y*_3_)	
Source	F	*P* value	F	*P* value	F	*P* value
Model	3.81	.0801	1.08	.4838	169.68	**.0001**
*X_1_*	1.60	.2617	0.11	.7556	326.82	**.0001**
*X_2_*	1.82	.2349	0.70	.4419	1.15	.3325
*X_1_X_2_*	0.21	.6682	3.33	.1277	13.97	**.0135**
*X_12_*	0.014	.9103	0.61	.4685	263.25	**.0001**
*X_22_*	1.21	.3209	0.53	.5011	0.77	.4199
*X_12_X_2_*	0.30	.6061	0.19	.6826	8.83	**.0311**
*X_1_X_22_*	2.51	.1738	0.90	.3857	3.18	.1348

^(a)^Significant effect (*P* value <.5) of factors on individual responses are shown in bold; *t*_80%_ release: time of 80 percent of drug release; *X*_1_: drug-to-polymer ratio; *X*_2_: stirring speed.
